# Signal Generation, Acquisition, and Processing in Brain Machine Interfaces: A Unified Review

**DOI:** 10.3389/fnins.2021.728178

**Published:** 2021-09-13

**Authors:** Usman Salahuddin, Pu-Xian Gao

**Affiliations:** ^1^Institute of Materials Science, University of Connecticut, Storrs, CT, United States; ^2^Department of Materials Science and Engineering, University of Connecticut, Storrs, CT, United States

**Keywords:** brain machine interface, brain computer interfaces, microelectrodes, neuron, signal processing

## Abstract

Brain machine interfaces (BMIs), or brain computer interfaces (BCIs), are devices that act as a medium for communications between the brain and the computer. It is an emerging field with numerous applications in domains of prosthetic devices, robotics, communication technology, gaming, education, and security. It is noted in such a multidisciplinary field, many reviews have surveyed on various focused subfields of interest, such as neural signaling, microelectrode fabrication, and signal classification algorithms. A unified review is lacking to cover and link all the relevant areas in this field. Herein, this review intends to connect on the relevant areas that circumscribe BMIs to present a unified script that may help enhance our understanding of BMIs. Specifically, this article discusses signal generation within the cortex, signal acquisition using invasive, non-invasive, or hybrid techniques, and the signal processing domain. The latest development is surveyed in this field, particularly in the last decade, with discussions regarding the challenges and possible solutions to allow swift disruption of BMI products in the commercial market.

## Introduction

**FIGURE 1:**
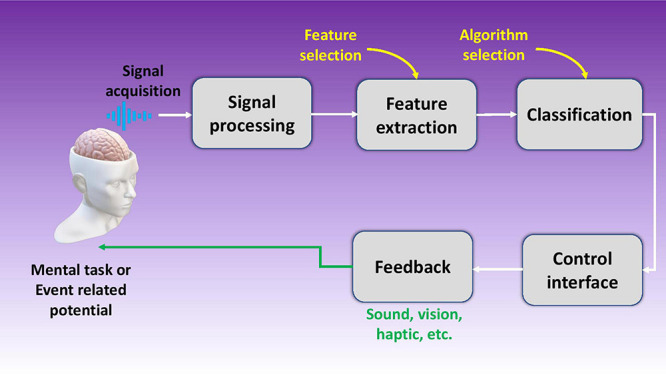
Schematic illustration of a brain machine interface using five stages including signal acquisition, signal processing, feature extraction, data classification, and the control interface.

Brain machine interfaces (BMIs) or brain computer interfaces (BCIs) are devices that connect our brains directly to computers. These intelligent systems can decipher brain signals using five consecutive stages: signal acquisition, pre-processing, feature extraction, classification, and control interface as shown in Figure 1 (Nicolas-Alonso and Gomez-Gil, 2012). Furthermore, BMIs can be either classified as motor, sensory, and sensorimotor, or they can be categorized as invasive or non-invasive depending upon which part of the brain they tap into or which part they are implanted in respectively. Today, an average computer processor can solve up to 1.8 billion calculations per second (cps) (Moravec, 1998), while human brain (1000 trillion; Mead and Kurzweil, 2006) can exceed this number by far and wide. It is noted the current interfaces being used to connect to the digital world, such as typing or voice commands, have very low bandwidth and throughput which hinders the market disruption of commercial BMI products. Hence there is a great need for better interfaces with higher bandwidth for seamless data transfer between our brains and computers.

To understand the need of BMIs/BCIs research and development, let us brief on their relatively young history and their initial development. For a detailed history of BMI, one may refer to the article by Lebedev and Nicolelis (2017). The research in this area started as early as 1950s, when Lilly (1956) implanted multielectrode array in a monkey’s cortical area for electrical stimulation. Later, Wyrwicka and Sterman (1968) recorded brain signals in cats which they later translated into sensory feedback for the same animals to increase the generation of those brain signals (sensorimotor rhythms). Around the same time, first electroencephalography (EEG) recording in humans were made to monitor the human brain activity (Butts, 1978). The term “Brain Computer Interface” was first coined by Vidal (1973), who successfully converted brain signals into computer controlled signals. By 1990s, Nicolelis and Chapin mastered one dimensional neural control of robotic limbs using laboratory rats (Chapin et al., 1999). The same group later worked on robotic arm control and developed BMIs for locomotion patterns and bimanual movement. Most recently, Donoghue et al. (2007) implanted invasive multielectrode arrays on humans to show BMI control of computer cursor (Hochberg et al., 2006) and robotic manipulator (Hochberg et al., 2012).

**FIGURE 2:**
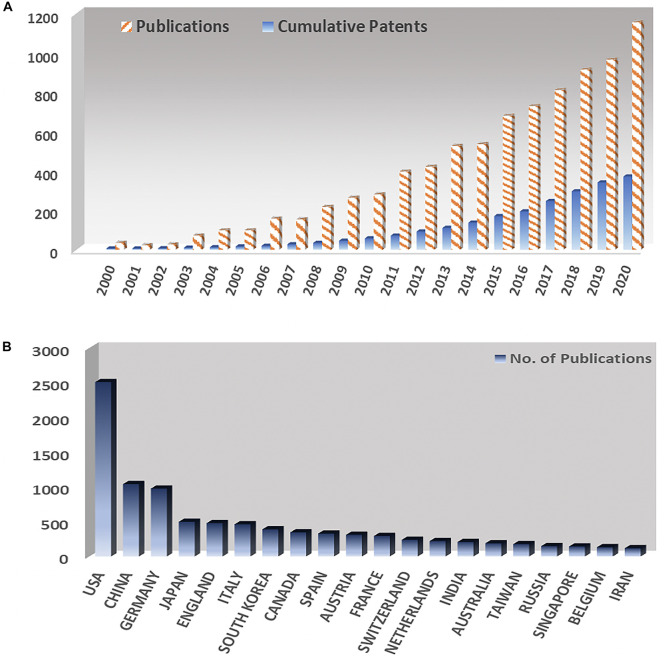
**(A)** Yearly number of publications and number of patents in the field of brain machine interfaces since 2000. **(B)** Number of publications on brain machine interfaces at different countries.

The most coveted application of BMIs to this date centers on developing assistive products for disabled people. It is noted that BMIs have tremendous potential to solve a variety of clinical disorders. Up till now more than 40,000 people have been successfully implanted with cochlear implant, a neuroprosthetic implant to restore audition in deaf people. Development and implant of BMI products such as speech synthesizers (Anumanchipalli et al., 2019), robotic prosthetics (Hochberg et al., 2006), and neuroprosthetic devices (Hochberg et al., 2012) have been successfully demonstrated by various research groups and companies using a limited number of neural electrodes. In the last 20 years, the interest in this field has been ever rising as evidenced by the trends in journal publications and patents as illustrated in Figure 2A. Currently, more than 100 research groups are actively working in the field of BMIs as compared to only three research groups 20 years ago (Wolpaw, 2007b). In the meantime, many companies are currently working in this field, such as Ni20, Neuracle, NeuroSky, Brain Co., SOSO H&C Co., Multineurons, Nihon Kohden Co., Neurable, Neuralink, and Bit Brain Technologies. Figure 2B shows an interesting trend of world leaders in the field, with United States leading ahead.

Despite the huge number of research groups and companies working in this field, surprisingly, very few BMI products are making it to the commercial market as of now. This review therefore is intended to shed some lights on those underlying reasons and steps that could be taken to aid in the development of efficient BMIs. In short, the field currently faces several challenges including the accuracy and efficiency issues of BMI products, among many others. For example, mental imagery based BMIs have only an accuracy of up to 75%, which means the system is mistaken once every four guesses (Allison and Neuper, 2010). Channel capacity, throughput or in simple words information transferred per unit time is still low for invasive BMIs and even lower for non-invasive ones. The throughput is up to 0.5 bits/s for non-invasive EEG based BMIs, whereas less than 3 bits/s for invasive BMIs. To put things into perspective, a simple task such as human tap (i.e., intentional finger tap on a surface) requires 10 bits/s of throughput (Baranauskas, 2014).

Meanwhile, the literature on BMIs is still very limited. There are indeed many reviews on neuronal function (Boniface, 1998; Dong and Benveniste, 2001; Del Bigio, 2010), neuronal recordings (Cheung, 2007; Ghane-Motlagh and Sawan, 2013) and processing of the extracted signal (Wolpaw, 2007b; Nicolas-Alonso and Gomez-Gil, 2012) but they all focus on specific parts of BMIs. Nevertheless, the intricacies and challenges would be worth surveying specifically at the intersection of different areas within the field. As such, the main purpose of this review is neither to detail BMIs’ history nor to expound upon its applications or products, instead intended to provide the readers with two distinct perspectives. One is to analyze the bigger picture as to how different areas within the field of BMIs amalgamate, and the other to detail the specific challenges and bottlenecks in each area and their possible solutions.

**FIGURE 3:**
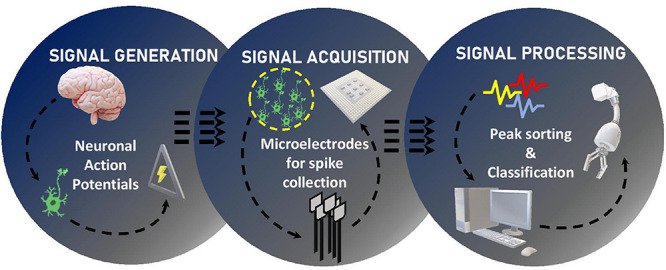
Schematic outline of this review on the three main domains in the research and development of brain machine interfaces.

Figure 3 shows the basic outline of this review, which includes three different sections, each describing and analyzing a particular area and challenges associated with it. The first section focuses on surveying the brain itself, how neurons work to generate signals, and other types of important cells that perform different functions within the brain. The second section details BMI types, methods, microelectrodes, and their limitations. Major microelectrode insertion problems are identified, such as buckling, corrosion, motion induced injuries, with the possible solutions to solve them discussed. The last section reviews signal post-processing methods and their limitations. Issues like low throughput and questionable data fidelity are also brought to light, due to limited usage of statistical analyses techniques.

## Signal Generation

Our brains are enclosed by pia mater, arachnoid mater, and dura mater beneath the skull, which are there to protect and support the brain. Human brain can be divided into three main parts based on function, i.e., the stem or cerebellum that deals with vital bodily function, the limbic system that deals with emotions, fight or flight response, and lastly the cortex that is responsible for processing, critical thinking, and analysis. The cortex can be further divided into frontal, parietal, occipital, and temporal lobes. A few major functions of these lobes include critical thinking, sensing and movement, memory storage, and visual data processing (Boniface, 1998).

The first theory on how the nervous system works was given by Camillo Golgi, who suggested in his reticular theory that there exists a continuous nerve cell network or reticulum. The theory was later rejected when Cajal and Sherrington proposed their model of neurophysiology which is valid and widely accepted till date (Cimino, 1999). The neurophysiology theory suggests that the nerve cells are discrete entities which communicate with each other using specialized contacts, so-called synapses. Nervous system is divided into two parts function-wise, i.e., central nervous system (CNS) which includes the brain and the spinal cord, and periphery nervous system (PNS) that includes sensory neurons which links sensory receptors present within the body at various locations (e.g., skin) to the CNS. Similarly, nervous system cells can be divided into two categories, neurons capable of electrical signaling, and the supporting glial cells, although not capable of electrical signaling but performing many of other necessary functions. A neuron is a specialized kind of cell that differs in many ways to a normal cell despite some similarities. There are many different types of neurons found within the human brain that are dedicated for different functions, still, a neuron primarily includes three parts namelydendrites, cell body, and axon. The dendrites, also called dendritic processes, often experience extensive arborizations which are the primary input source from other neurons at the synapses. These dendrites are equipped with high content of cytoskeletal protein and ribosomes that help in information receiving and processing. However, there exist a few types of neurons without dendritic parts, thus with limited input capabilities accordingly (Wood, 1996; McCandless, 1997).

The synapse comprises of pre- and postsynaptic components called presynaptic terminal and postsynaptic specialization, respectively. However, there is no cellular continuity between these elements for most of the synapses that occur. Instead, an extracellular space exists in between called the synaptic cleft that is used by the neurons to communicate using specialized molecules called neurotransmitters. There can be as many as 100,000 synaptic inputs received by each neuron in human brains. The integrated signal after passing through the cell body is read out at the axon origin, which is the part of the cell responsible for signal conduction. These axons can range from a few microns in length, usually found in interneurons, to almost a meter, found in spinal cord transmitting signals to distant regions of the human body. The whole activity of this electrochemical signaling within a neuron is called action potential (AP) (Buzsáki et al., 2012). So basically, the dendrites, body, and axon are responsible parts of a neuron for information receiving, processing, and transferring, respectively (Purves et al., 2008).

**FIGURE 4:**
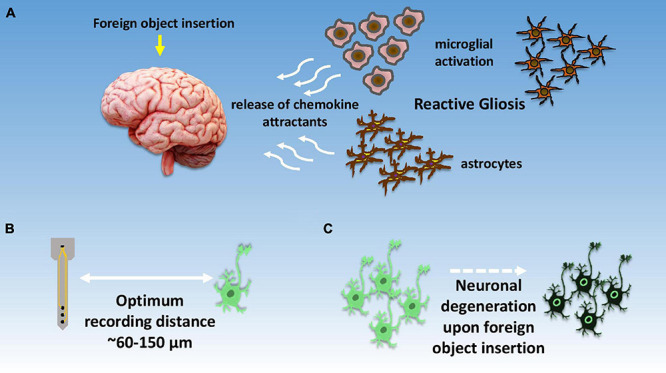
Illustrative summary of neuronal response toward signal recordings. **(A)** Depiction of foreign body response within the brain and initiation of reactive gliosis due to local brain inflammation; **(B)** optimal distance for recording neuronal spikes with high signal to noise ratio, **(C)** localized neuronal degradation after electrode insertion within brain tissue.

Glial cells, on the other hand, do not directly take part in the synaptic interactions, and outnumber neurons by three times. The word glia is of a Greek origin which means “glue,” based on the assumption that these cells hold the neurons together, however, there is little evidence on that. Glial cells perform many important functions such as maintaining the ionic balance within the brain, supporting injury recovery, providing scaffold for neural development, and controlling neurotransmitters around synaptic cleft. There are primarily four types of glial cells found in the CNS, namely, astrocytes, oligodendrocytes, microglia, and ependymal cells. Astrocytes having star-like elaborate processes are responsible for maintaining a suitable chemical environment for neural signaling as shown in Figure 4A. Oligodendrocytes do the job of axon myelination in the CNS to help in faster signal transfer. The same job is done by Schwann cells in the PNS. Microglia performs the function of cellular debris removal from injury sites. They also secrete a variety of cytokines to modulate local inflammation. In a way, their function is very similar to immune cells or macrophages. Lastly, ependymal cells are responsible for the production of cerebrospinal fluid (CSF). Figure 4B depicts the optimum signal recording distance for a single neuron (Buzsáki, 2004) while Figure 4C pictorially represents the degradation of neurons upon foreign object insertion in the brain.

### Foreign Body Response

As mentioned in the introduction, understanding the functions of different types of brain cells is crucially tied to the development of efficient and long-lasting BMIs. Hence, it is necessary to discuss here how these above-mentioned cells respond to a foreign object such as a synthetic electrode in the context of BMIs. It is known that human body actively responds to any foreign object/device implanted in it, which is so called foreign body response (FBR). Generally, the body responds in five stages including (i) initial injury upon implantation, (ii) foreign body-tissue interaction, (iii) acute inflammation, (iv) chronic inflammation, and (v) foreign body enclosure in fibrous capsule (Morais et al., 2010). Nevertheless, the brain takes a slightly different approach for tackling any foreign body mainly due to the presence of blood brain barrier (BBB) and absence of immune cells that are otherwise present everywhere in the human body. This does not mean that the brain is incapable of responding against foreign bodies. To address this issue, brain has glial cells that are assumed to be almost ten times as abundant as neurons (Hilgetag and Barbas, 2009), however some studies suggest the population of neurons and glial cells is almost the same (Azevedo et al., 2009). As mentioned earlier, microglia, astrocytes, oligodendrocytes, and ependymal cells are the major types of glial cells present in the CNS (Matthews, 1976). Together these cells are responsible for many critical activities including protection against infection, maintain homeostasis, axon myelination, and CSF secretion, etc. (Baumann and Pham-Dinh, 2001; Dong and Benveniste, 2001; Hanisch and Kettenmann, 2007; Del Bigio, 2010).

Upon electrode insertion in the brain, a severe damage to surrounding tissues and BBB disruption occurs. This activates microglia and astrocytes from a resting state to an active state to initiate the healing process. The whole process is called reactive gliosis. The active states of microglia and astrocytes enhance their proliferation and begin secreting various chemokine attractants. Studies suggest that within 30 min of electrode insertion, microglial lamellipodia initiate implant encapsulation. In the next 24 h, activated microglia completely encapsulates the implant, while astrocytes begin migration toward the affected site and commence astrogliosis and glial scaring (Stence et al., 2001; Babcock et al., 2003; Sofroniew and Vinters, 2010; Kozai et al., 2014). It takes almost 2–3 weeks for the astrocytic encapsulation in contrast to the microglial encapsulation which occurs within the first 24 h of implantation, while reorganization of blood vessels follows glial scaring (Stichel and Müller, 1998; Szarowski et al., 2003).

**FIGURE 5:**
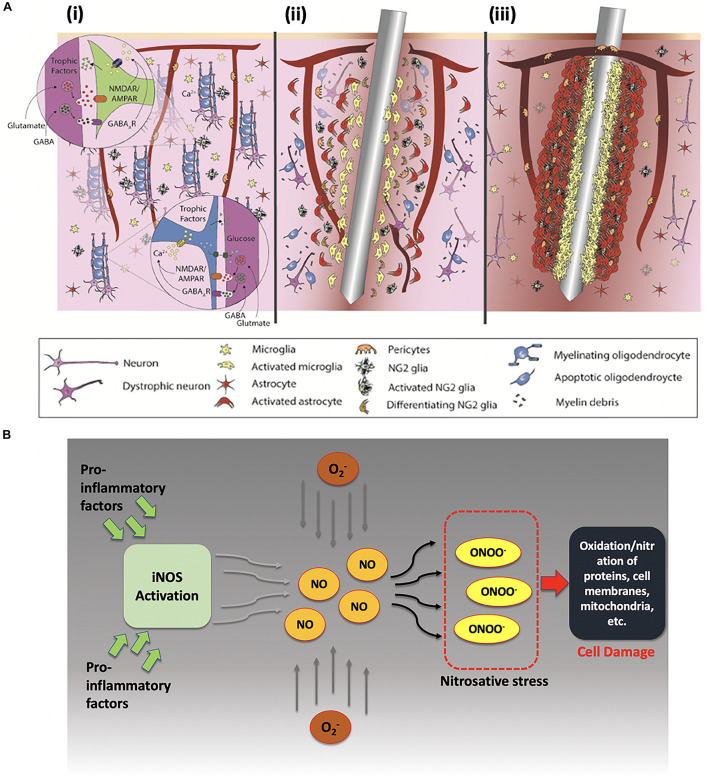
**(A)** Foreign body response upon electrode insertion. (i) Healthy brain tissue, (ii) acute response upon electrode insertion within 6–8 h, and (iii) chronic response after injury persisting for months. Reprinted with permission from Wellman and Kozai (2017). Copyright (2017) American Chemical Society. **(B)** Effects of proinflammatory factors on neuronal cells after electrode insertion. Proinflammatory factors activate inducible-nitric oxide synthase (iNOS) that produces nitric oxide (NO) in excessive amounts, which later reacts with reactive oxidative species to form peroxynitrite (a known marker for cell damage).

Figure 5A depicts how the brain responds upon the insertion of an electrode. Figure 5A (i) shows in healthy brain tissue glial cells provide neuronal modulation and neurotrophic support by releasing GABAergic (GABA-gamma-aminobutyric acid) and glutamatergic neurotransmitters. However, electrode insertion sets forth a series of biochemical events near the injury site. Due to electrode shank penetration, capillaries and cellular processes are severed, extracellular matrix is disrupted, and platelets and erythrocytes are released. Microglia, NG2 glia, the only glial cells that receive direct synaptic input form neurons, and astrocytes get activated experiencing behavioral andmorphological changes. Activated microglia migrates toward the electrode surface to encapsulate it, while releasing proinflammatory factors, such as cytokines and chemokines, at the same time. Pericytes get detached from vasculature and rush toward the injury site for repairing broken vasculature as shown in Figure 5A (ii). Astrocytic encapsulation proceeds microglial phagocytosis as depicted in Figure 5A (iii). The entire process also causes neurodegeneration as well as demyelination of axons due to the death of oligodendrocytes in the vicinity at the brain-electrode interface (Jorfi et al., 2015; Wellman and Kozai, 2017; Ferguson et al., 2019).

Moreover, under such inflammatory conditions, the release of proinflammatory factors occurs and leads to production of nitric oxide via inducible NO synthase enzyme and other reactive oxygen species (ROS) like superoxide ions. Upregulation of ROS has also been reported under proinflammatory conditions as shown in Figure 5B (Chan, 2001). These reactive ions then react to form peroxynitrite and other reactive nitrate species (RNS). Under these nitrosative and oxidative stress conditions, RNS and ROS can interact with proteins, lipids, and mitochondrial components which may lead to eventual cell damage (Choi, 1993; Cherian et al., 2004).

The FBR is also especially important to study because of its chronic nature. The initial acute response alleviates within 6–8 days of electrode insertion. However, the prolonged chronic response persists even after several months (Chan, 2001). Several studies prove that if the electrodes are inserted and quickly removed, there are no signs of electrode tracks after a few months, suggesting that the presence of electrode within the brain exacerbate the proinflammatory conditions probably due to FBR and micromotion (Polikov et al., 2005; Griffith and Humphrey, 2006).

## Signal Acquisition

**FIGURE 6:**
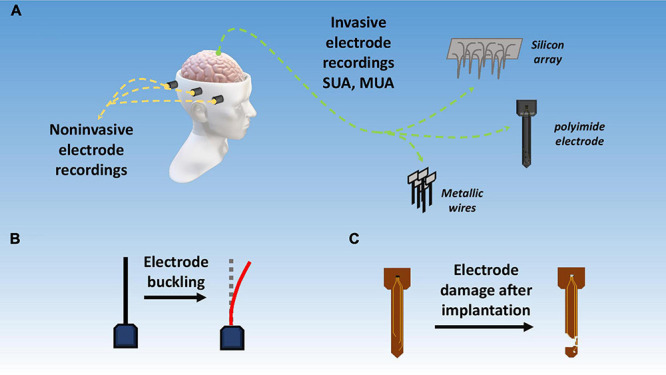
Signal acquisition techniques and challenges. **(A)** Invasive vs. non-invasive BMI techniques; **(B)** electrode buckling upon intracortical insertion; **(C)** damage to electrode due to corrosion, passivation layer degradation, and mechanical mismatch.

**FIGURE 7:**
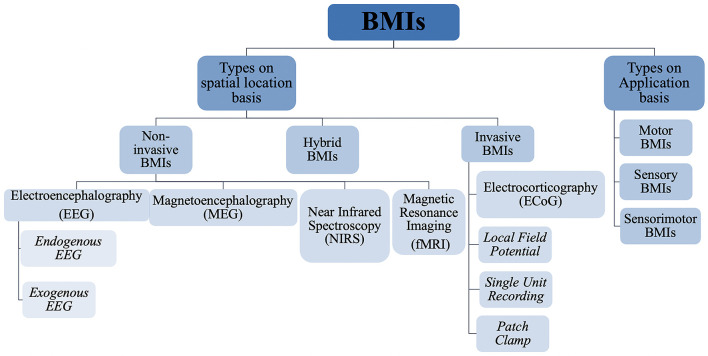
Hierarchical classification of brain machine interfaces based on their spatial position and application.

Based on invasiveness, BMIs can be divided into two main categories, namely, invasive, and non-invasive BMIs as shown in Figure 6A. The major difference between these two techniques is that invasive techniques require surgery to implant electrodes within the brain’s cortex while non-invasive techniques rely on recordings over the skull. Generally non-invasive methods have poor spatial resolution but show reasonable temporal resolution. Also, signal attenuation is a big problem in such techniques due to limited electrical conductivity of skull (Van Gerven et al., 2009). Recently, another class of BMIs has also emerged, utilizing the benefits of both invasive and non-invasive techniques, appropriately called as hybrid BMIs (Pfurtscheller et al., 2010). Elaborated in below sections are the most common types of these three techniques with a focus on the invasive techniques. Figure 7 gives a hierarchal classification of BMIs.

### Non-invasive Techniques

One of the most used neural recording techniques is EEG, in which electrodes are simply placed on the surface of scalp at specific points to record averaged neuronal signals from different intracortical regions (Schomer and da Silva, 2012). EEG based systems are portable and are usually cheap. They have good temporal resolution as they directly measure the neural activity while it lacks in spatial resolution as the signal has to pass through a number of physical barriers including skull, scalp, and CSF (Wolpaw, 2007a; Babiloni et al., 2009). Also EEG recordings are susceptible to artifacts that can be mechanical, electromyographic, or electrooculographic in nature (Fatourechi et al., 2007). Magnetoencephalography (MEG) is another technique which records postsynaptic activity of neurons using magnetic fields. Its spatial resolution is reasonably better than EEG and has a high temporal resolution (Cohen and Halgren, 2009). Functional magnetic resonance imaging (fMRI) is a method used widely in medical science to create 3D maps of brains. It basically detects the changes in magnetic field occurring due to changes in oxygenation levels of hemoglobin due to neuronal activity (Lee et al., 2009). The signal generated by fMRI is also called “blood oxygen level dependence” (BOLD) (Ogawa et al., 1990). It can be used to obtain full brain scans covering all brain areas unlike EEG or MEG (Logothetis et al., 2001).

Other than using electrical signals, neural data can be obtained using photons in the wavelength range of 650–900 nm that can penetrate cortical areas and show contrasts based on oxygenation/deoxygenation of hemoglobin. The method is called near infrared spectroscopy (NIRS) (Owen-Reece et al., 1999). Functional near infrared topography (fNIRT) is another modification of NIRS that renders 3D images of the brain (Limongi et al., 2009). Some other known methods include positron emission tomography (PET), single positron emission computed tomography (SPECT), and computer axial tomography (Herman and Brouw, 1982; Lassen, 1987; Ollinger and Fessler, 1997).

Several evoked or induced responses are being used to obtain neural response via non-invasive techniques. These include visually evoke potentials, slow cortical potentials, sensorimotor rhythms, event related potentials, event related synchronization/desynchronization (ERD), blood oxygenation levels, and cerebral oxygenation levels, etc. (Coyle et al., 2003). Table 1 show types of brain signals recorded using non-invasive BMIs.

**TABLE 1:** Types of brain signals recorded using non-invasive BMIs.

**Signal type**	**Origin site**	**Frequency (Hz)**	**Time interval (s)**	**Throughput (bits/min)**
Slow cortical potential (SCP) (Birbaumer, 1999)	Frontocentral region	0.5	0.5–10	5–12
Sensorimotor rhythms (Pfurtscheller and Aranibar, 1979)	Somatosensory and motor cortex region	8–13	0.25–0.5	3–35
P300 event related potential (P300-ERP) (Rivet et al., 2009)	Parietal lobe	>6	300 × 10^–3^	20–25
Steady state visually evoked potential (SSVEP) (Lesenfants et al., 2014)	Occipital cortex	12–18 (Kuś et al., 2013)	500–1000 × 10^–3^	60–100

Non-invasive techniques are widely used and well established, however, the major shortcomings of almost all the non-invasive techniques are low signal specificity, low signal to noise ratio (SNR), and signal distortion. The hindrance due to skull and intermediate brain layers between the cortex and the electrodes reduces the SNR of the recordings, leading to an average signal of millions of neurons. Moreover, any of the above-mentioned techniques cannot record a single or even a few hundred neurons, which is highly critical for practical BMI applications. Hence, a logical step forward for obtaining a specific high-resolution signal is to put electrodes directly outside or inside the cortex.

### Invasive Techniques

**FIGURE 8:**
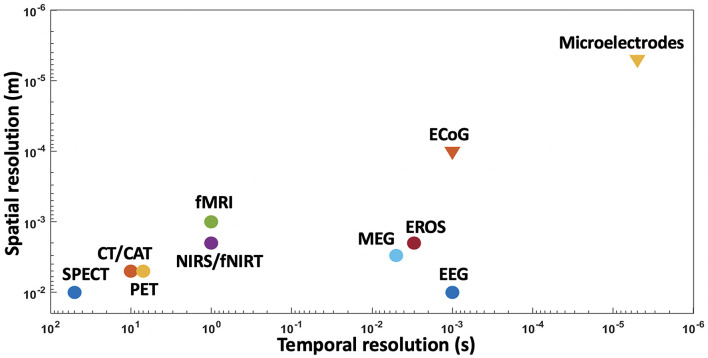
Spatial and temporal resolutions of various BMI techniques, with microelectrodes showing higher resolutions than other techniques.

As mentioned earlier, non-invasive techniques have been used in the past to develop neuroprosthesis but their signals are distorted, nonspecific, and low-resolution (Stence et al., 2001; Babcock et al., 2003). To precisely record neuronal data with a higher degree of freedom for neuroprostheses, the development of BMIs will require invasive recording techniques (Lebedev and Nicolelis, 2006) such as electrocorticography (ECoG) and intracortical electrodes. Figure 8 clearly depicts that the invasive techniques have far better spatial and temporal resolutions than non-invasive techniques.

To our advantage, the SNR is relatively higher in invasive techniques. ECoG requires surgery to place electrodes in extracortical areas either inside or outside dura mater, called subdural ECoG, and epidural ECoG, respectively (Schalk et al., 2007). This technique is like EEG but with a higher SNR as the electrode grid is placed directly above the cortex surface avoiding skull. ECoG records an average of thousands of neurons, it can also be referred as local field potentials (LFPs) but is unqualified to obtain deep brain signals (Kaiju et al., 2017). However, AP readings from a group of functionally linked single neurons are required for high precision and increased data fidelity (Yuste, 2015). To achieve this, microelectrodes are used to record single unit activity (SUA) as well as multi-unit activity (MUA). Though even with SUA, a specific number of neurons must be recorded to derive some consistent and trustworthy meaning from the readings. Opinions vary, but a good estimate for minimum number of readings can be anywhere between 15 and 30 neurons (Nicolelis and Lebedev, 2009). Hence, intracortical SUA and MUA recordings using microelectrodes are very important.

#### Microelectrodes

This section proceeds with the discussion of microelectrodes, including their types, materials for fabrication, and specific issues related to such electrodes. Table 2 illustrates the importance of reducing electrode size for BMIs. First, the size reduction usually provides us with a higher SNR. To obtain good quality signal, electrodes are required to have good charge transfer characteristics, low impedance, and high selectivity (Keefer et al., 2008). Macroscale electrodes have very low selectivity and can cause severe damage to brain, which limits their use in BMI applications.

**TABLE 2:** Critical properties of different sized electrodes.

	**Macroelectrode**	**Microelectrode**	**Nanoelectrode**
Area (mm^2^) (Finot et al., 2003)	2	1.25 × 10^–3^	6.4 × 10^–4^
Peak current (μA)	5.1	7.5 × 10^–3^	45 × 10^–3^
Signal to noise ratio (SNR)	2.5	6	70

**FIGURE 9:**
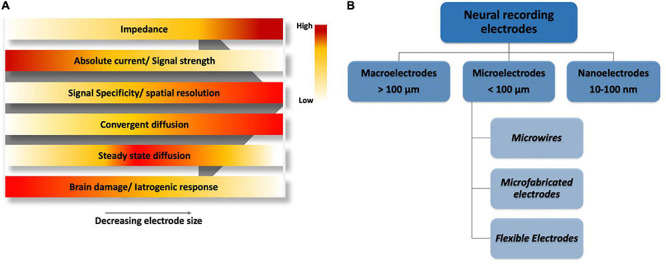
**(A)** A qualitative electrode performance comparison based on size, horizontal axis shows a decreasing electrode size ranging from macroelectrodes on left, microelectrodes in the middle and nanoelectrodes on far right. **(B)** Classification of electrodes based on size.

Figure 9A qualitatively shows that as selectivity is increased by reducing the size of the electrode, impedance also increases (Rui et al., 2012), hence, microelectrodes give us the optimum size range with high quality signal. Meanwhile, active species transport is affected by the size of the electrode. Although convergent diffusion increases as the electrodes get miniaturized, non-fickian diffusion becomes competent due to the brownian motion of molecules which in turn affect the steady state current. Moreover, as theabsolute current decreases proportionally with the electrode size, it gets harder to separate signal from noise if the current is too low as in the case of most nanoelectrodes. In addition, electrode handling and implantation at nanoscale is also a challenge. With a relatively optimum size range, microelectrodes are of suitable features such as small capacitive charge current, high current density, and low impedance (Szunerits and Walt, 2002).

Figure 9B shows the classification of electrodes based on their dimensions. Macroelectrodes are typically larger than 100 μm and in the millimeter range, while nanoelectrodes are between 10 and 100 nm. In contrast, microelectrodes have at least one dimension less than 100 μm (Compton et al., 2008). Microelectrodes can be broadly classified into three types, namely, microwires, micromachined electrodes, and flexible electrodes depending upon the material used for their fabrication.

#### Microwires

Microwire electrodes are a type of microelectrodes that dates back to 1940s when these electrodes were first used for electrophysiological studies (Renshaw et al., 1940). They have the capability to record highly localized extracellular potentials of nearest neurons exposed to the tip of the electrodes, with the rest of the electrodes insulated (Loeb et al., 1995). Metal wire electrodes show high SNR due to low impedance and can be used for single cell recordings, or as an array of microwires to record a number of neurons (Low, 1976; Chapin, 2004). Three major issues are associated with metal wire electrodes, including the wire bending during implantation, limited recording duration, and incompatibility with silicon-based electronics (Cheung, 2007; Prasad et al., 2014).

Renshaw et al. (1940) conducted one of the earliest experiments that used Ag/AgCl electrode for direct recording of electrical signals from single neurons. During the same time period use of other metals such as platinum, tungsten, gold, iridium, and stainless steel was also recorded (Grundfest and Campbell, 1942). In the later years, electrolytic pointing was used to fabricate sharpened stainless steel microwires that in turn reduced electrode variability and tissue damage (Grundfest et al., 1950). Although these early microwire-based electrodes had shorter recording spans, ranging from hours to a few days, they proved to be extremely useful in the development of early BMIs and helped scientists understand collective behavior and memory forming patterns of neurons (Evarts, 1966). In 1968, a platinizing process was used to coat electrode tips with spongy platinum black which enhanced the active surface area of electrodes to improve SNR (Robinson, 1968). Stainless steel provides good signal quality but is fragile near the electrode tip, hence, tungsten was used as a substitute, having higher strength and toughness than stainless steel. But tungsten has its own drawbacks such as signal distortion at low frequency range. Platinum is another excellent candidate for wire electrodes, giving a high SNR, stable signal, and low impedance. The only problem with platinum, like stainless steel, is its mechanical fragility. Some studies have shown that iridium is also a suitable material for microwires as it is stiff, corrosion resistant, and provides a high charge density (Grundfest et al., 1950; Hubel, 1957). During the last decade, attempts have been made to coat microwire tips with carbon nanotubes and polymers to enhance performance and reduce impedance (Gross et al., 1985).

Microwires can be used to successfully record or stimulate single neurons and an array of microwires can be used to record a sizeable neuronal population. Hence, microwires can be used for deep brain stimulation to treat epilepsy, paralysis, and Parkinson’s disease (Chapin, 2004). Lastly, microelectrodes have issues such as wire bending during implantation, short recording spans, and sometimes incompatibility with silicon-based electronics (Cheung, 2007).

#### Micromachined Electrodes

In 1959, the advent of lithographic techniques widened the possibility window for neural recordings. Silicon, a biocompatible material that is also suitable to etch integrated electronics on it, proved useful to develop Michigan and Utah type electrodes. The Michigan-type electrode was developed at University of Michigan in 1980s and comprised of a penetrating tine with a sharp tip exposed for recordings. Whereas, Utah-type electrode is comprised of sharpened, conductive silicon needles in a matrix (Wise et al., 1970; Campbell et al., 1991). A typical 10 by 10 grid of Utah array electrodes is 4.2 mm wide and 1.2 mm in length (Maynard et al., 1997). Unlike microwires that can move apart after or during insertion in the brain, micromachined electrodes stay fixed relative to each other and relative spatial positions are determined during the micromachining process. Another advantage of these electrodes is their compatibility and integration with circuitry, signal processing and wireless interfaces. Therefore, silicon is an excellent material, for theseelectrodes, due to its strength, biocompatibility, and integration feasibility with other circuitry (Ghane-Motlagh and Sawan, 2013).

Microfabricated electrodes are made in a series of carefully controlled steps. In Michigan-style electrode, a thermal oxide mask is used to etch a boron stop on silicon wafer to define substrate dimensions. Then, a dielectric layer is added for back side insulation. Later, conduction traces are formed on the top surface to connect bonds pads to recording sites. Recording sites and bond pads are then formed using gold or other metals. Conducting traces are finally insulated and the shanks are coated with polymers like Parylene-C for enhanced protection during *in vivo* recordings (Jorfi et al., 2015). Utah-type electrode uses a different fabrication approach. Glass reflow, etching, and dicing techniques are used to create a Utah electrode array (UEA). UEA is widely used and approved by United States Food and Drug Administration. The tips of silicon needles/tines in UEA are further coated with platinum or iridium oxide while the rest of the parts are insulated using a polymer. Individual channels are further isolated using glass dielectric between bond pads (Jorfi et al., 2015).

#### Flexible Electrodes

The most recent type of electrodes is based on flexile materials, mostly polymers to address the strain and inflammation issues in the stiff and rigid silicon and metal-based electrodes. Fabrication of such soft electrodes is very challenging to ensure biocompatibility and high SNR. Especially the *in vivo* insertion of such electrodes is quite difficult. Several techniques are used to insert soft electrodes such as shuttles, insertion robots, and self-degrading materials.

During the last decade, polyimide based flexible electrodes have been developed with good biocompatibility, mechanical flexibility, resistance to solvents, and a reasonable SNR (Cheung et al., 2007). Another polymer, Parylene has been used as a substrate to fabricate electrodes that are flexible and show a high SNR with stable electrical contact (Metallo et al., 2011). Flexible CNT based electrodes have alsoshown reduced impedance and high charge transfer capability for neural recording applications (Chen et al., 2011). SU-8, a negative photoresist, is another mechanically and chemically stable material that has been used for flexible electrode fabrication (Del Campo and Greiner, 2007). Similarly, Polydimethylsiloxane (PDMS), an elastomer, can also be used for flexible electrode microfabrication. Moreover, Polypyrrole (PPy), Polythiophene (PTh), and Polyaniline (PANI) are a few of the many conductive polymers being used in this decade for flexible electrodes (Lee et al., 2016).

Recently, Neuralink has shown the successful synthesis of highly flexible electrode arrays, with each array containing up to 96 threads and each thread containing 32 independent electrodes, using polyimide as substrate and gold thin film traces. These threads are 5–50 μm in width, 20 mm in length and up to 6 μm thick. In order to reduce impedance and increase charge carrying capacity of these miniaturized electrodes, they are coated with poly-ethylenedioxythiophene doped with polystyrene sulfonate (PEDOT:PSS) (Musk and Neuralink, 2019). With the advancement in coating and microfabrication technologies, such flexible electrodes seem to be the future of microelectrodes as they have considerable advantage over metal and silicon-based electrodes.

#### Microelectrodes’ Implantation Issues

Now a fair base for microelectrode types and materials has been established. It is fitting to detail some prominent issues with the insertion of these microelectrodes in the cortex. As described in the “Foreign Body Response” section of the article, the brain reacts strongly to any foreign object that enters it. Microelectrodes are no exception. Some of the specific issues with microelectrodes insertion are discussed in this section.

Recording stability, signal quality deterioration and electrode performance degradation over time are still the major hurdles in neuronal recordings using microelectrodes. Delisle Burns et al. (1974) showed an electrode dysfunction of as high as 92% occurred in just 5 months of unit recording in a cat’s cerebral cortex, as a result of the neuroinflammatory response of the brain as described above. Efforts to alleviate brain cells inflammation and injury to vasculature as well as cells can help in getting a stable signal from electrodes for long durations. Many factors, as discussed below, are responsible for early degradation of neuronal signals.

Microelectrodes can face several issues during and after *in vivo* implantation. These issues can range from purely mechanical in nature, like stiffness mismatch and buckling, to chemical like corrosion and passivation layer degradation (Barrese et al., 2013). Also, after the implantation, the brain can suffer injury due to micromotion of the electrode within the cortex, prolonging neuroinflammation.

#### Stiffness Mismatch

A good estimate for the stiffness of a material can be Young’s modulus which is a ratio of stress to strain. Thelesser the difference between the Young’s modulus of the material to that of the brain tissues, the better it is. Silicon, for example, has a modulus of 190 GPa, whereas the modulus for gray matter in the brain is just ∼3 kPa (Elkin et al., 2007; Hopcroft et al., 2010). Obviously metals, silicon or even glass based materials are million times stiffer than the soft brain tissues and hence they evoke vigorous inflammatory response in the brain. This very fact propelled the development of softer flexible electrodes to reduce stiffness mismatch. Several polymeric materials such as PDMS, polyimide, Parylene C, and PTFE have Young’s modulus in the range of a few MPa to a few GPa but still the difference of stifness as compared to brian tissues is staggering, as shown in Table 3. Morever, the softer the material, the harder it is to insert it in the brain without buckling, avoiding the vasculature.

**TABLE 3:** Young’s moduli of different electrode materials in comparison with brain tissues.

	**Material**	**Young’s modulus**
Brain tissues	Collagen/gelatin	2–200 MPa
	PEG	0.2–2 GPa
	Silk	1.7–2.8 GPa
	Brain’s gray matter (Budday et al., 2015)	2.2 kPa
	Brain’s white matter (Elkin et al., 2007)	3.08 kPa
Candidate electrode materials	Glass (Hand and Tadjiev, 2010)	60–100 GPa
	Tungsten (Qiu et al., 2002)	410 GPa
	Silicon (Hopcroft et al., 2010)	190 GPa
	Polycarbonate	2–2.4 GPa
	Steel	200 GPa
	PTFE	0.5 GPa
	Rubber	0.01–0.1 GPa
	LDPE	0.11–0.86 GPa
	Polyimide	4 GPa
	Parylene-C	2.8 GPa
	Polydimethylsiloxane (PDMS) (Sun et al., 2004)	∼1 MPa

**FIGURE 10:**
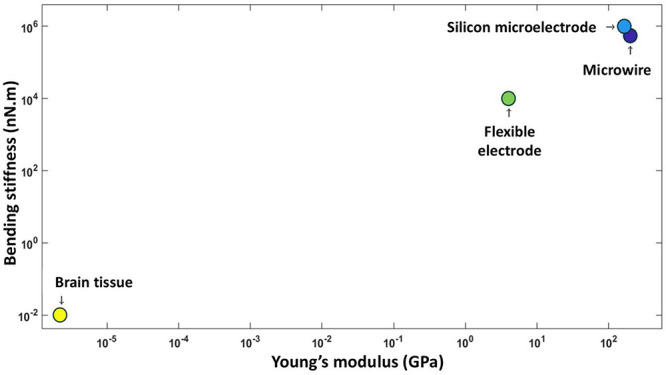
Mechanical properties of brain tissue and three commonly used electrode materials.

Figure 10 makes clear the difference between bending stiffness and Young’s modulus of existing neural electrodes and brain tissues (Fu et al., 2017). The silicon electrode is microfabricated using lithography techniques while the microwire and flexible electrodes are made from stainless steel 304 and polyimide, respectively. Average bending stiffness and Young’s modulus values are taken form literature to draw the graph.

#### Mechanical Damage

Although not a big concern, mechanical damage of electrodes, shanks, tines, and other components of the implants still needs some attention. Recent reports show evidence of mechanical damage to parts of the recording system during or after implantation as shown in Figure 6C (Prasad et al., 2014; Fu et al., 2017). Brittle materials show higher tendency towardfailure hence the use of tougher more flexible materials is recommended.

#### Buckling

Buckling is an issue more relevant to softer and flexible materials including both polymers and metals. Buckling means the bending or warping of the electrode during an intracortical insertion as shown in Figure 6B. During electrode insertion, three types of forces act on the microelectrode, i.e., an axial tip force, a frictional force on electrode surface, and a compressive clamping force. Together these forces can be called as total insertion force (TIF) and it should be greater than 1000 μN to circumvent buckling (Egert et al., 2011). Buckling force itself can be given by Euler’s formula.


(1) $$F_{buckling}=\frac{\pi^{2}I_{x}E}{{\left(KL\right)}^{2}}$$

where *I*_*x*_, *E*, *K*, and *L* represents moment of inertia, modulus of elasticity, effective length factor, and length of the electrode, respectively. The compressive force required for an electrode to buckle is directly proportional to the product of Young’s modulus and moment of inertia (i.e., second moment of area) and inversely proportional to the length of the electrode. For a given material Young’s modulus is constant. Hence, the only two parameters that can be change are the cross-sectional area or the length of the electrode.

Common materials currently being used, although having high bending stiffnesses, stillsuffer from buckling problem. One way to tackle this issue is to increase the width or thickness of the electrodes but that will in turn increase the initial iatrogenic injury and neuroinflammatory response (Wester et al., 2009). Another approach is the use of reinforced polymers at the expense of increased Young’s modulus as evident from the study conducted by Lee et al. (2004). Some studies have shown the use of shuttle to avoid buckling (Capadona et al., 2005; Ereifej et al., 2013). Microfluidic channels with in the electrodes is a novel methods used by Takeuchi et al. (2005). Dissolvable PEG was filled in the channels to provide reinforcement. These microfluidic channels can also be used for drug delivery and other sensing operations. One innovative way to stop buckling isto make electrodes in the form hollow tubes to increase the moment of inertia and hence increase the force required for buckling. But, if the thickness of the tube is very small, which is desirable, the electrode will suffer from brazier buckling, a local crumpling of the electrode at various points along the length of the electrode. One last solution to buckling can be inspired from nature, specifically bamboo trees which have nodes along the length of the bamboo to provide structural integrity. Similar techniques can be implied to synthesize electrode with nodes at regular intervals to enhance structural integrity against buckling (Gordon and Stewart, 1980).

#### Corrosion

Corrosion is a big problem for stimulating electrodes in which electrochemical reactions can occur under applied electric field. But corrosion can also be observed in non-stimulating/recording electrodes due to faradic charge transfer over time. Prasad et al. (2012) reported structural changes in microelectrode over time. Corrosion is strongly dependent on material. For example, tungsten is a material that can easily be corroded in saline solution under non stimulating conditions.

Moreover, the surrounding environment can also accelerate the rate of corrosion. During brain’s inflammatory response, ROS are produced surrounding the area of implant due to FBR. ROS show a positive correlation with corrosion as an increased corrosion was observed for tungsten surrounded by oxidative oxygen species (Patrick et al., 2011). ROS also facilitate neurodegeneration. Materials such as tungsten that form a passivation layer to resist corrosion or platinum that performs excellent in saline solution and also reduce the concentration of reactive oxidative species, by converting hydrogen peroxide species to water, are excellent contenders for corrosion resistant microelectrodes (Ishigami, 1998; McCarthy et al., 2011; Potter et al., 2014).

#### Passivation Layer Degradation

Microelectrodes are coated with different passivation layers for insulation and corrosion resistance. However, these passivation layers have the tendency to degrade over time which in turn affects the performance of the electrode itself. Silicon oxide, one of the materials used to fabricate microelectrodes, shows *in vitro* stability for more than 21 months, it surely degrades in the *in vivo* scenario (Winslow et al., 2010). A recent study showed that silicon carbide offers no measurable dissolution at 30°C. Use of polymer-based insulation is a recent common practice although whether it actually enhances recording longevity is still a topic open for debate (Lecomte et al., 2018). Certain neuron adhesion materials like poly-D-lysine or laminin in conjunction with anti-inflammatory agents like dexamethasone or alpha-melanocyte can block the production of nitric oxide to reduce inflammation (Staii et al., 2009; Feng et al., 2018; Dey and Bishayi, 2019). One study showed degradation of polyimide-based insulation on tungsten electrodes as early as 42 days after implantation (Prasad et al., 2012). Another study proclaimed that there was no difference in the neuroinflammatory response of coated vs. uncoated electrodes (Loeb et al., 1977).

#### Motion Induced Injury

One possible reason of prolonged neuroinflammation can be the motion induced injury within the brain, pertaining to mechanical mismatch of electrodes and brain tissues. *In vitro* studies support the micromotion hypothesis, however, little to no *in vivo* data is available in this domain. One factor further exacerbating the strain on brain tissues is the tethering of microelectrode devices to the skull. Tethered implants generates a much higher FBR than the untethered ones, as shown in one study (Biran et al., 2007).

Recently, a collaboration in the fields of molecular genetics and photonics has rendered a new field named optogenetics. This is a highly researched field these days and it uses light activated proteins called opsins, injected in target neurons, to stimulate the neurons to modulate brain activity with the temporal accuracy of milliseconds (Yizhar et al., 2011).

Researchers are trying to find novel and more effective ways to develop more advanced BMI technologies. In this effort, Kim et al. (2010) developed a silk based electrode mesh that can be theoretically rolled up and inserted on top of the brain, eventually adjusting itself to the gyrus and sulcus of the brain surface (Kim et al., 2010). Yeo et al. (2013) used a similar technique to print electrode array onto human skin and it is expected to extend this technology to human brain too. Another group has demonstrated the use of a neural mesh in which electronics are encapsulated into a free standing conducting polymeric mesh that can be inserted into the brain using a syringe (Liu et al., 2015). Seo has demonstrated the design of a novel concept for BMIs called neural dust. The design involves very tiny silicon sensors (100 μm) spread inside the cortex which communicate with a subdural transceiver placed above pia meter to retriever brain signals using ultrasound technology (Seo et al., 2013).

### Hybrid BMIs

A hybrid BMI is comprised of one BMI and another system (which could be another BMI). Utilizing more than one interfaces usually helps in improvement of overall performance and reduction of false positive. A qualifying condition for hybrid BMIs is that at least one of the signals should directly be recorded from the brain. A typical hybrid BMI has more than one inputs, which could either be processed simultaneously or sequentially.

Simultaneous hybrid BMIs can either use one brain signal corresponding to two different mental strategies (e.g., EEG signal from spatial visual attention and motor imagery), two different brain signals (e.g., intracortical electrical signal and hemodynamic response), or one brain signal and another input that does not pertain to brain specifically. Foundational work by Claude Bernard proves mutual action and reaction between the brain and heart due to pneumogastric (vagus) nerve. Tumati et al. (2021) and Pfurtscheller et al. (2021) have also highlighted brain heart coupling by reporting neurocardiac desynchronization in anxiety disorders and change in vascular and neural BOLD oscillations due to anxiety, respectively. Hence, an electrocardiogram (ECG) based switch can act as an additional input for the hybrid BMI with another brain signal based input (Thayer and Lane, 2009). These additional input signal can also come from electrooculogram (EOG) recording for eye gaze tracking or electromyography (EMG) for muscle electrical response tracking (Punsawad et al., 2010; Zander et al., 2010; Leeb et al., 2011).

In sequential systems, one system can act as a switch for the activation or inhibition of the other system. The purpose of such a brain switch is to recognize a specific pattern in brain during ongoing brain activity. Such a switch should not produce any false positives without user intent or will. The first EEG based brain switch was developed by Mason and Birch (2000). However, these switches are not restricted to EEG and high amplitude threshold SSVEPs or hemodynamic changes measured using NIRS can also be incorporated to develop a brain switch (Cheng et al., 2002; Coyle et al., 2007).

## Signal Processing

**FIGURE 11:**
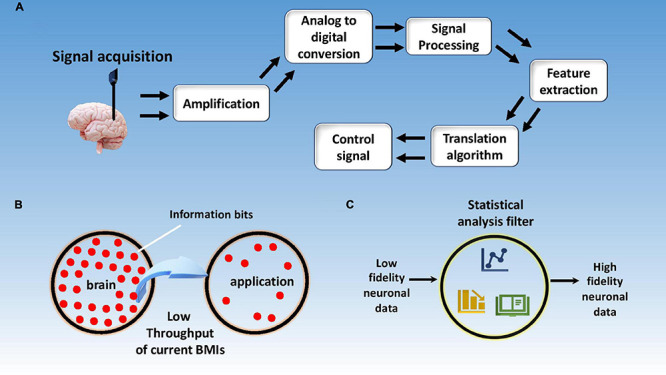
Signal processing schematics and challenges. **(A)** Schematics of signal processing steps involved in neural data decoding; **(B)** current BMIs having a limited data transfer rate form brain to application; **(C)** neural data with high functional dimensionality need go through a rigorous statistical analysis to avoid false positive results.

**FIGURE 12:**
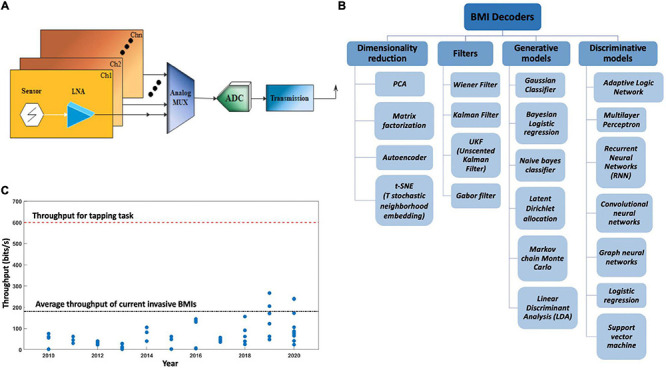
**(A)** Schematics of a brain machine interface’s electrical circuit. LNA, low noise amplifier; MUX, analog signal multiplexer; ADC, analog to digital converter; Ch, channel. **(B)** Different types of BMI decoders used in signal post processing. **(C)** Information throughput over the years, blue dots represent the throughputs of invasive and non-invasive BMIs as reported in different published articles over the years.

Figure 11 depicts the summary of this section. Independent of the type of microelectrodes used, Figure 12A depicts the basic electrical circuit used for electrical stimulation and recordings for single neurons. First, the collected signal is passed through a low noise amplifier (LNA) to allow noise reduction and increase the SNR. The stack may also include (Coyle et al., 2007) a signal multiplexing unit to reduce the cable requirements in tethered systems. After the multiplexing signal is digitized using an analog to digital converter (ADC), spike sorting may also be done in the same stack to reduce wireless data transfer volume. This preprocessed data is then sent to a computer wirelessly for neural decoding (Chestek et al., 2009).

Brain’s electrical activity can be recorded intracellularly or extracellularly depending upon the position of the electrode. Extracellular activities of neurons that can also be called APs are transmitted along the axon in a frequency range of 100 Hz to 10 kHz, for a duration of a few milliseconds. The amplitude of these APs can range anywhere between 50 and 500 μVpp based on the proximity of the electrode tip to the recording site (Mollazadeh et al., 2009b). As described earlier, the LFP recordings record the averaged potential of several neurons in the vicinity of electrode tip. The first step after recording neuronal activities is to amplify it. The amplified signals are later passed through low pass filter to get LFPs. Further, these can be high pass filtered using sorting algorithms and spike detection to get SUAs. For high resolution SUAs, electrodes should be located between 50 and 100 μm from the neurons of interest. Any neurons between 100 and 150 μm can still be recorded but with a relatively low SNR and are regarded as MUAs. Anything beyond that is considered noise (Buzsáki, 2004; Leeb et al., 2011).

Different neural activity patterns can be observed in recorded data which translates to different thinking activities. A very challenging task is to extract meaning from these patterns for which these patterns are categorized into classes based on their features (Pesaran et al., 2018). Brain signals are generated with in a highly noisy environment and APs of neurons of particular interest are always overlapped in space and time by other neurons firing at the sametime within the same domain due to several different brain activities. Brain signals are inherently non-stationary and time dependent. Signals can be sometimes divided into smaller segments to reduce power and processing requirement but usually at the expense of decreased accuracy. One way to avoid this is to apply dimensional reduction techniques to the data thereby removing redundancy and unimportant parts. Principal component analysis and independent component analysis are two of the prominent techniques for achieving this.

A BMI decoder is a system that applies a transform algorithm to neuronal inputs to calculate output variables. BMI decoders used for feature extraction and classification employ a variety of statistical and machine learning techniques. These decoders particularly fall under the class of multiple input multiple output (MIMO) models (Kim et al., 2006).

Linear decoders are a type of decoders that use a weighted average of neuronal rates to calculate output variables. Georgopoulos, one of the few proponents of these kind of decoders, proposed a population vector theory for neuronal signal decoding. He suggested that neuronal activities can be expressed as vectors with preferred direction being the one with the maximal firing rate (Georgopoulos et al., 1986). Weiner filter is another linear decoder that uses an approach based on reducing the mean square error.

Another type of decoder very similar to Wiener filer is Kalman filter (White et al., 2010; Gilja et al., 2012) that uses a discrete time step approach to predict output variables. A variation of Kalman filter is the unscented Kalman filter (UKF) to deal with nonlinear data (Li et al., 2009). Some other point process models apply a probabilistic function to predict neuronal spiking based on certain known parameters (Lawhern et al., 2010). Artificial neuronal networks is one of the most recent techniques for neuronal data decoding (Chapin et al., 1999). Figure 12B lists different types of BMI decoders used for BMI applications based on their utility.

Furthermore, brain activity characterization can be done either through classification or through regression. In regression, extracted features are treated as independent variables to predict user will, while classification methods use features as independent variables to draw limiting boundaries for decision making. Classifiers usually suffer from a dimensionality problem. The number of training data sets needed foroptimum results increase exponentially with the dimensionality of the feature vector. This is sometimes referred as the dimensionality curse. Also, bias-variance tradeoff is another problem. Ideally, low bias with low variance are desired but when one is lowered, the other increases (Jain et al., 2000; McFarland and Wolpaw, 2005). Classifier overfitting is another issue that needs to be dealt with.

### Signal Processing Issues

Like electrode insertion related issues as discussed in the neural recordings section, the methods and techniques used to process the neural data also have some shortcomings. Some of the problems can be avoided using better classification techniques or sophisticated statistical analyses methods but quite more than often a tradeoff needs to be made between one or the other desired property as mentioned in the above section about variance and bias.

#### Noise Removal

A high quality, low noise signal ultimately translates to a BMI with high efficiency and accuracy. An archetypal brain signal can be represented as (Semmlov, 2004; Kawala-Sterniuk et al., 2021);


(2) B(t)=S(t)+N(t)

*B*(*t*), *S*(*t*), and *N*(*t*) are measured bio-signal, actual deterministic signal, and additive noise, respectively. Signal noise is also another issue that distorts the signal and needs to be eliminated or reduced to obtain clear neuronal spikes. It is noted that motion-induced artifacts such as blinking, eye movement, skull movement can add to the distortion of recorded signal and need be accounted for in the techniques used for signal filtering (Zúquete et al., 2010).

More specifically, the first step after fetching the signal from brain is amplification. On average, neural signals acquired from electrodes have amplitudes ranging from 50 to 500 μm. Due to electrochemical effects at the electrode-tissue interfaces, these signals suffer from DC offsets across different electrodes, which have a magnitude of 1–2 V, significantly larger than the signal itself. Extracellular APs have frequencies ranging from 300 Hz to 8 kHz. Hence, it is a difficult job to filter noise while keeping the signal intact. A number of different groups have reported LNAs but most of them have some limitations that still need to be addressed (Mollazadeh et al., 2009a; Waldert et al., 2009).

#### Information Throughput Limitation

In addition, information throughput is one of the biggest limiting factors in the advancement of BMI applications. Typical EEG based BMIs and commercial invasive BMIs have an information transfer rate of up to 0.5 bits/s and <3 bits/s, respectively (Semmlov, 2004; Klobassa et al., 2009; Kawala-Sterniuk et al., 2021). To put things into perspective, a simple human tapping task requires a transfer rate of ∼10 bits/s (Fitts, 1954) while a slow human speech would require ∼7–15 bits/s (Brown et al., 1992). It reflects that any complex human task will require an information rate higher than 10 bits/s. Moreover, the unit of transfer rate, bit, is defined on a logarithmic scale hence the difference between 10 and 3 bits/s is several orders of magnitude. Figure 11B displays low information throughput from brain to application, while Figure 12C shows reporting of throughput for BMIs over the years by different research groups globally.

#### Data Fidelity

Lastly, with the availability of a plethora of signal processing and data collection techniques at hand, risk of false positive also increases. In fact, Bennett et al. (2009) published their results, showing the perils of deriving erroneous results due to inapt usage of statistical methods. The problem is prevalent in brain signal data classification due to a high functional dimensionality of neuronal data. It was shown that the probability of false positive is quite high across 130,000 voxels in a typical fMRI volume (Bennett et al., 2009). The publication also informs us about the fact that a considerable portion of neuroimaging articles do not use any statistical buffers to check the soundness of their derived results.

Moreover, the accuracy of the acquired brain signal is dependent upon a number of factors like dimensionality of the data, signal noise, interference from other signals, information transfer rate, etc. (Dornhege et al., 2007). Accuracy of signal is highly important in functional BMI applications and is one of the many bottlenecks in this field. Figure 11C represents the concept of increasing the reliability of neuronal data by apply statistical filters to the recorded data.

#### User Training

Most of the current classification methods require a lot of training for class differentiation. User training is a time consuming step and the efficiency of the classifier depends a lot on these training sessions. A tradeoff is usually made between the number of training sessions conducted and understanding the complexity of brain signals. The most important parameters that affect the trainings efficacy are the level of difficulty, training duration, environment, instruction, open loop training vs. closed loop (with online feedback), real or virtual, self-paced vs. synchronous (system-paced) training, etc. (Roc et al., 2021). A few articles mistakenly relate the limited reliability of BMIs to user incompetency, labeling it as “BCI illiteracy” (Vidaurre et al., 2011; Blankertz et al., 2010). However, it does not at all mean that the users are the poor performers, it actually point toward the fact that our currentuser training protocols are inappropriately designed (Lotte et al., 2013; Thompson, 2019).

Brain machine interface training approaches can be briefly critiqued at three different levels, namely, feedback, instructions provided to user, and training tasks (Lotte et al., 2013). Feedbacks should be supportive, non-evaluative, timely and specific, rather than just indicating the correctness of a task (Shute, 2008). Multimodality can also be introduced in feedbacks in comparison to conventional unimodal feedbacks, however, its usefulness is still debatable (Ainsworth, 2006; Merrill, 2007). Instructions provided to the user for a specific task are hardly given any importance, whereas, it is known that feedback tends to be more effective with clearly defined instructions (Neuper et al., 2005; Hattie and Timperley, 2007). Also, engaging and involving tasks, as opposed to synchronous and repeated tasks, enhance the efficiency of computer mediated learning (Reeves and Nass, 1996; RRyan and Deci, 2000).

User training is a trivial obstruction that hinders the disruption of BMIs in the market. This is a cumbersome step that the user must go through before actual utilization of the product. The smaller and easier the training step is, easier the adoption of BMIs becomes. Most of the current BMI signal decoding techniques treat frequency time and spatial dimensions separately to predict the user intent. Models that deal with interdependencies of these characteristics can significantly improve BMIs’ functionality. Another very crucial thing that will aid toward the commercialization of BMIs is unsupervised adaption. Such adaptive classification algorithms are still in nascent stages and are not at all fit to be employed for commercial BMIs (Nicolas-Alonso and Gomez-Gil, 2012).

Moreover, user training especially plays a significant role in mental task-based BMIs (MT-BMIs). Research efforts in the field of MT-BMIs have led to improvement in signal processing algorithms and machine learning approaches. These approaches are mostly based on classifiers that are either trained offline on collected user data or online for co-adaptive MT-BMIs (Lotte et al., 2007). One of the advantages of this co-adaption is the reduced user training time and flexibility for user, as the user does not have to adapt to a fixed system. Based on fixed learning rates, research also shows that online classifier training produces better results than offline classifier training (Millan, 2004). Monto et al. proved the existence of a slow cortical excitability cycle, possibly controlled from structures within brain stem, hence, presentation of task (e.g., motor imagery) in such subject specific intervals may help shorten the training time (Monto et al., 2008; Pfurtscheller et al., 2020).

## Discussion and Outlook

Brain machine interfaces, first developed in 1950s, have seen tremendous improvements and breakthroughs in the last two decades. This review highlights three major domains that comprise BMIs, namely, signal generation, signal acquisition, and signal processing. Fundamental concepts, major challenges, and bottlenecks in these three domains have been delineated. We believe that a large audience base can benefit from this review as it is not specific to any one domain or field and gives a broader outlook on BMIs and apt overarching layout of this field.

Deeper understanding of signal generation and transmission within the brain can help in development of more efficient BMIs. Complete understanding of FBR in brain is also required to invent and modify existing electrode technology. As detailed in this review, BMI development switched (Hattie and Timperley, 2007) from metal microwires to silicon based micromachined electrodes (Pfurtscheller et al., 2020) with the advent of lithographic techniques. Recently, interest has shifted toward flexible conductive polymer-based electrodes to better match the mechanical properties of the brain and to limit the neuroinflammatory response of the brain.

Further investigation is needed in invasive electrode technologies to minimize tissue damage, increase long-term stability, and lower the risk of infection. Specifically, two material properties that dictates this mechanical behavior of electrodes are Young’s modulus and bending stiffness. Electrodes with relatively lower Young’s modulus and bending stiffness will limit the iatrogenic injury and neuro-inflammation. Although invasive signal recording technologies have advantage over non-invasive BMIs, they still have limitation and drawbacks. Hybrid BMIs can be one of the solutions incorporating the strength of both technologies while largely avoiding their drawbacks. But hybrid technology is still far from maturation and improvement in hybrid BMIs involving three or more signals is needed. Prominent issues related to hybrid BMIs like high cost, user workload and system complexity also need to be dealt with, to transition these hybrid devices to commercial market.

Furthermore, precise and more adaptable BMI signal decoders/classifiers are required to accurately decode user intent from the acquired signal. Understanding signal time, frequency, and spatial dimensional interdependencies can play a crucial role to achieve this goal. Most of the current BMIs are still at lab scale and still needs improvement in all three above mentioned domains to introduce them in commercial market. Prominent factors hindering their commercial scale disruption are low information throughput, low reliability, low accuracy, steep learning curve, and fear of invasive electrode insertion. Information throughput can be increased by adopting hybrid technology while accuracy and reliability can be enhanced through iterations in product development and incorporation of statistical filters in signal processing stages. Unsupervised learning could be the most significant step toward commercial disruption of BMIs but there are still a lot of challenges that should be tackled, as previously discussed in user training section of this review.

Brain machine interfaces are not only limited to medical application for disabled people but are widely applicable in domains like education, entertainment, sports, neuroimaging, behavioral understanding, etc. One of the main limitations of this work is that it does not cover all the new findings and relevant literature spanning all three domains. It is practically impossible to do that due to plethora of ever-increasing available literature. However, a broad, widely applicable account has been presented showing relation between different domains and intra-domain challenges with a reasonable focus on important details. Research community is encouraged to take inspiration from this work to address major challenges in their respective domains while considering the bottlenecks and challenges in other relevant domains discussed in this review.

Several private companies have come forward in this decade, promising commercial products in the near future. The number of research groups working on BMIs has also exponentially increased in the past two decades. This might suggest that an age of commercial BMI products is not far from realization. This could significantly change the way we interact with computers and machines marking the inception of a new era of human-machine symbiosis that could eventually help humans achieve insurmountable goals.

## Author Contributions

P-XG and US contributed to perceive and design the project and, read and approved the submitted version. US wrote the first draft of the manuscript. P-XG revised and finalized the manuscript. Both authors contributed to the article and approved the submitted version.

## Conflict of Interest

The authors declare that the research was conducted in the absence of any commercial or financial relationships that could be construed as a potential conflict of interest.

## Publisher’s Note

All claims expressed in this article are solely those of the authors and do not necessarily represent those of their affiliated organizations, or those of the publisher, the editors and the reviewers. Any product that may be evaluated in this article, or claim that may be made by its manufacturer, is not guaranteed or endorsed by the publisher.
